# Playing the genetic lottery: an interview with Kiran Musunuru

**DOI:** 10.1242/dmm.050508

**Published:** 2023-10-10

**Authors:** Kiran Musunuru

**Affiliations:** Perelman School of Medicine, University of Pennsylvania, Philadelphia, PA 19104 USA


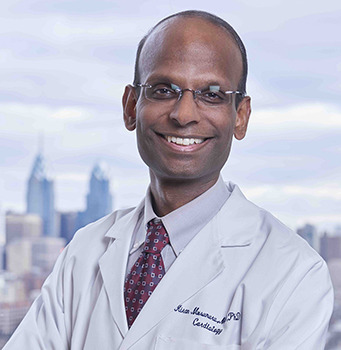


**Kiran Musunuru.** Photo credit: Peggy Peterson of Peggy Peterson Photography.

Professor Kiran Musunuru is a principal expert in genetic research and medicine. As a physician scientist and active cardiologist, his research has been primarily centred on the investigation of cardiovascular diseases. Throughout his career, he has pioneered large-scale human genetic studies and applied emerging gene editing tools to interrogate the mechanisms of disease in model systems, with the ultimate goal of developing innovative gene editing therapies.

Kiran did his PhD in biomedical science at The Rockefeller University before studying medicine at Weill Cornell Medical College. He is now based at the University of Pennsylvania, where he is the Director of the Genetic and Epigenetic Origins of Disease Program and the Scientific Director of the Center for Inherited Cardiovascular Disease. He serves on the National Heart, Lung, and Blood Institute Advisory Council of the National Institutes of Health and on the Board of Directors of the American Society of Human Genetics. His ground-breaking research has been widely recognised as he is the recipient of many prestigious awards, including the Presidential Early Career Award for Scientists and Engineers from the White House, the American Heart Association's Award of Meritorious Achievement and Joseph A. Vita Award, the American Philosophical Society's Judson Daland Prize for Outstanding Achievement in Clinical Investigation, and the American Federation for Medical Research's Outstanding Investigator Award. In this interview, we discuss some of his most exciting research, as well as the future of genetic research and gene editing therapies.

**To begin with, what discovery or research project have you found most exciting in your career?**

I care a lot about cardiovascular disease, because it is the world's leading cause of death, affecting 18 million people a year worldwide. Even in the poorest countries on Earth, cardiovascular disease has become the leading cause of death, making it the preeminent global health threat of the 21st century. I'm a little biased, because I'm a cardiologist, but nonetheless, I think objectively you can say that's true. I'm particularly excited by an ongoing study that involves individuals who inherited genetic factors that give them very low levels of low-density lipoprotein (LDL) cholesterol and protect them from cardiovascular disease. Before 2009, a family had been identified with very low LDL cholesterol levels, but efforts to identify the causal gene were unproductive, simply because of limitations in the technology. In 2009, exome sequencing changed the game because we could easily sequence all coding regions of the genome. We applied exome sequencing to individuals in the family with the most pronounced phenotype and found that four siblings had two different nonsense mutations in the same gene, causing complete knockout of angiopoietin-like 3 or *ANGPTL3* (Musunuru et al., 2010).

Around the same time, we were doing a genome-wide association study (GWAS) with 100,000 individuals – I think the biggest that had been done at that time – on blood lipid traits, including LDL cholesterol, high-density lipoprotein (HDL) cholesterol, triglycerides and total cholesterol (Teslovich et al., 2010). We found 95 loci associated with these traits, and one of these loci was *ANGPTL3*, which had been robustly associated with LDL cholesterol levels. So, we had two studies with very different approaches that converged on the same target, providing concordant lines of evidence that ANGPTL3 was in fact, an important regulator of LDL cholesterol.

Then, the real question was, can we really prove that loss of functional ANGPTL3 not only reduces LDL cholesterol levels, but also protects against cardiovascular disease? The very existence of four healthy siblings who entirely lack ANGPTL3, have lived to an old age, have never had any heart disease or any other serious diseases, and have had children who are healthy, speaks strongly (Stitziel et al., 2017). This supported the idea that if you lack ANGPTL3, you've won the genetic lottery and you are protected against cardiovascular disease. Additionally, there's a village in Italy that came to light where there was a reasonably high prevalence of individuals who had one or two copies of the same *ANGPTL3* mutation (Noto et al., 2012). As far as we can tell, they're totally healthy. Then, analysing large databases, we found that about one in 300 people in the general population have one variant copy of *ANGPTL3* (Stitziel et al., 2017). We then introduced the human gene with these different variants into a *Angptl3* knockout mouse model, which allowed us to easily and rigorously classify variants as neutral or loss-of-function (Stitziel et al., 2017). It then became clear in our human population study that loss-of-function mutations in *ANGPTL3* are protective against cardiovascular disease. This made it that much more of a compelling therapeutic target to treat diseases associated with high LDL cholesterol.

Familial hypercholesterolemia is a genetic condition that causes very high LDL cholesterol levels, most commonly due to mutations in the LDL receptor. The LDL receptor is on the surface of basically every cell in the body but found in particularly high concentration on the surface of hepatocytes in the liver. The problem is that if patients lack the LDL receptor, then some of the more classic ways of reducing LDL cholesterol don't work and these patients rely on a dialysis-like procedure once a week to clean the cholesterol from their blood. However, we found that ANGPTL3 regulates LDL cholesterol independently of the LDL receptor, which means that you can target this gene in patients with very high cholesterol who don't have any LDL receptor (Gaudet et al., 2017).

It's a beautiful example of harnessing a population bell curve: we've studied people at one extreme of the bell curve, with very low LDL cholesterol, to directly inform the treatment of patients at the other end of the spectrum.

Now, the question is, how do you turn off *ANGPTL3* in these severely affected patients? No one has identified a small-molecule solution to inhibiting ANGPTL3, but because it is made in the liver and secreted into the bloodstream, where it has a lot of action, you can make a monoclonal antibody against it. The company Regeneron did exactly that and it was approved last year for use in patients with familial hypercholesterolemia (Dewey et al., 2017; Mullard, 2021). It's exciting that our identification of a drug target led to a US Food and Drug Administration (FDA)-approved drug that is now being used in patients, only 10 to 11 years later.

However, my interest is asking whether we can take these protective mutations in *ANGPTL3* and put them into people to help them that way. The answer is CRISPR genome editing. We've been focused on developing CRISPR-based therapies to target genes like *ANGPTL3*. It's working well in mice, as we can deliver CRISPR into the liver – which is where ANGPTL3 is made – to introduce loss-of-function mutations, which leads to around a 95% reduction of the protein (Chadwick et al., 2018; Kasiewicz et al., 2023). We've done this in monkeys too, which has all worked really well (Kasiewicz et al., 2023), and we're aiming for early-phase clinical trials in 2024. This is the dream of any physician scientist, to be able to bring things full circle by starting at the bench, and then taking it all the way to the bedside. It's a beautiful example of harnessing a population bell curve: we've studied people at one extreme of the bell curve, with very low LDL cholesterol, to directly inform the treatment of patients at the other end of the spectrum.

[…] if I can't deliver CRISPR into the heart muscle itself, which we really can't do yet, then that's not something I should be putting all my energy into. Instead, I see incredible opportunities to tackle a whole variety of liver-centred diseases.

**You have a master's degree in law – why did you choose to study this and how does it influence your research?**

Taking gene editing to the clinic requires studies in large animals and a lot of work to check boxes before a regulatory agency will allow you to move into early-phase human clinical trials. It's very hard to do that in the academic setting, so in 2017, we decided that taking the *ANGPTL3*-targeting gene therapy to the clinic would require a much larger, well-funded effort, which basically means industry. So, my colleagues and I co-founded Verve Therapeutics™. We had to obtain funding and licence technologies, which touches upon the law, and this was my primary motivation to start taking classes in law. The University of Pennsylvania Law School had launched a Master in Law programme not intended for people who wanted to practice law, but people who, in their professional careers, were interacting with the legal system in various ways. It ended up being incredibly useful because I learned about intellectual property law, business law and the lifecycle of companies. Administrative law is extremely important for drug development, because the regulatory interactions with agencies like the FDA require a lot of work and, more importantly, a lot of knowledge about how the system works. After my masters, I spent a year and a half almost full time at the company getting things going, before going firmly back into the academic setting.

That entire experience, both the legal education and the hands-on experience with the company, has shaped my thinking around therapy development. My initial focus was on genes like *ANGPTL3* and cardiovascular disease, but my academic lab has more recently pivoted to other diseases. This is because I realised that the door to the liver is wide open, in terms of gene editing. There are obviously big limitations remaining, but in principle, we know how to deliver different types of CRISPR technologies into the liver for gene editing. By targeting the liver, we could treat inborn errors of metabolism that cause enzyme deficiencies. At the top of the list is the disease phenylketonuria (PKU), for which newborn screening started in the 1960s. A lot of our effort over the last year has been developing gene editing technologies to correct the disease-causing mutations in patients with PKU. We've gotten a lot of traction with this, as it's been working well in cells and humanised mouse models (Brooks et al., 2023). We've received a $26 million grant from the National Institutes of Health to take the PKU gene editing treatment to the clinic. So now we're thinking about the next steps to take this approach to a regulatory agency and get approval to do human clinical trials. From my past experience, and from my education in law, I now think it's actually viable to do this in the academic setting.

It's been interesting for me, because I'm a cardiologist and I still take care of patients with cardiovascular issues, but my research focus has shifted to a different set of diseases. It's great to say that I want to wipe out all types of heart diseases, but if I can't deliver CRISPR into the heart muscle itself, which we really can't do yet, then that's not something I should be putting all my energy into. Instead, I see incredible opportunities to tackle a whole variety of liver-centred diseases. Maybe in a few years, we'll have definitive treatments for a lot of rare genetic disorders for which there are not currently good treatments. It's a very exciting place to be even though I've ventured out of my comfort zone of cardiology.

**You previously mentioned the power of GWAS, but what improvements need to be made in large-scale population studies for us to make the next leap in understanding and treating disease?**

GWASs have been a wonderful approach. We have learned so much about the genetic architecture of disease and identified literally hundreds or even thousands of loci that are associated with any particular trait or phenotype of interest. So, there's no question that they have enormous value. If you take a complex disease, like cardiovascular disease or atherosclerosis, what we now know, because of GWAS, is that there's a lot more to biological risk for heart disease than we appreciated even 10 years ago. We knew high cholesterol, high blood pressure and smoking were risk factors, but now we know that there are at least half a dozen or more categories of pathways or gene programmes that influence the risk of cardiovascular disease.

Now, the interesting question is, what is the practical value of all of this? When you go to a meeting like the American Society of Human Genetics Annual Meeting, there's a lot of excitement around polygenic risk scores. Cardiovascular disease and atherosclerosis were some of the very first traits in which polygenic risk scores were defined and then applied in an attempt to better understand and predict disease risk. It all comes back to that bell curve I mentioned earlier; if you have a polygenic risk score that is at an extreme of the bell curve, then it does seem to forecast either a much higher risk for disease or substantial protection against disease.

So, there's a lot of fervour to start applying polygenic risk scores that are directly informed by GWAS to clinical practice, but we're not quite sure of the path to get there yet. Part of this is because there was a big bias in the early days of GWAS towards populations of European ancestry, meaning that polygenic risk scores are heavily biased towards individuals of European ancestry. If we charge ahead and start putting this into clinical practice, then we know it's going to be more useful for certain people than others. In some populations, the polygenic risk scores, as they currently exist, will be entirely uninformative, and may even be counterproductive. There's not just the potential of having unequal benefits to different people, but there is potential to harm some people if you get it wrong. I think there's still a lot more work to do with respect to the polygenic risk scores and we clearly need more GWASs of much more diverse groups of people before we're ready for primetime.

My interest as a physician scientist is less focused on risk prediction, and more on identifying new therapeutic targets and developing new therapies. We now have very powerful means to do this with genome editing technologies that would have been unreasonable even 15 to 20 years ago. To do this, you need to figure out how novel genetic risk factors identified by GWASs actually influence disease, which is incredibly challenging. It took us a couple of years of really hard work in cells, mice and human cohort studies to understand just one locus out of what ultimately became hundreds or thousands of loci associated with cardiovascular disease. It's very satisfying to do that in an intellectual sense, but what we found at the end of the day is that biology is messy. The idea that you have this nice, clean therapeutic target ends up not being the reality for most of these loci identified by GWASs, especially for variants in non-coding regions. We've learned a lot of biology through GWASs, but through no fault of our own, we haven't done so well in identifying a large number of actionable therapeutic targets, which is what I think all of us hoped for back in the early 2000s, when the Human Genome Project was coming towards partial completion.

Where there has been more traction is exome-wide association studies, because the biology is cleaner. These studies identify rare loss-of-function variants in the coding regions of the genome that have a strong association with disease. You can accumulate data from large numbers of individuals, potentially including millions of people from diverse populations. This can identify a much cleaner therapeutic target. Then, with genome editing technologies, or more traditional therapeutic modalities, we can develop new drugs that will help patients with a whole variety of diseases.

With any therapy that you administer to the foetus, you have to worry about unintended consequences to the mother. That entails another level of complexity […]

**You have been exploring *in utero* base editing in mice to prevent congenital genetic disorders. What hurdles remain for this type of approach?**

I have to say up front that *in utero* or foetal gene editing is not the same as germline editing in an embryo. The motivations for foetal gene editing are rare genetic disorders that are already starting to cause damage at the foetal stage. With improvements in genetic technologies, and the ability to do prenatal genetic diagnosis through amniocentesis, or even analysis of blood samples taken from the mother, we're now able to diagnose these genetic disorders well before birth. The challenges in foetal gene editing are similar to the challenges in treating adults or children, which is delivery of the gene editing therapy to the correct organ system or part of the body. Foetal gene editing is more complicated because the patient is not easily accessible. One way to achieve this is to directly administer the gene editing therapy to the umbilical vein, which targets the therapy to the foetal liver quite readily (Rossidis et al., 2018). If you want to target the lungs, you can administer the gene therapy directly into the amniotic fluid and the foetus will actually breathe in this amniotic fluid as the lungs develop (Alapati et al., 2019). In preclinical animal models, it's been working quite well, and you can prevent a disease from happening before birth. It's pretty clear that if you can do that, there is an even better outcome than treating someone very early after birth.

The biggest challenge is that it's not just the patient whose safety you have to worry about. There's another very important entity involved – and that's the mother. With any therapy that you administer to the foetus, you have to worry about unintended consequences to the mother. That entails another level of complexity to the regulatory framework, in terms of sufficiently establishing safety to allow the first early-phase clinical trials to go forward. It's going to take a lot of work in small and large animal models, and a lot of discussions with regulatory agencies before we get to that point. But it'll come, I'm confident of that.

Any technology that has the potential for great use, also has the potential for great misuse.

**With these advancing technologies and therapeutic possibilities, how can we ensure medical progress is enabled in the most ethical way?**

That is a big challenge. The only way you can truly enforce things is through legislation and regulation, and that doesn't come from the scientific community, that comes from governments. As scientists and clinicians, we can hold ourselves to very high standards, make recommendations, inform advisory committees and panels, and produce very thorough comprehensive documents, but we're not in a position to enforce regulations on anyone. We can just urge, advise and even beg, but at the end of the day, it's up to politicians to decide whether they wish to legislate or regulate science to prevent untoward uses of these technologies. Any technology that has the potential for great use, also has the potential for great misuse.

The gene-edited embryo scandal is a very good example (Musunuru, 2019). Before it happened in 2018, there was a lot of debate and discussion in the community, and deliberate attempts were made to make these conversations inclusive of the scientific communities around the world to achieve a consensus about the responsible way to proceed. But then look what happens when scientists don't get the message and forge ahead in semi-secretive conditions. When the news broke, everyone was scandalised and horrified. It was a massive scientific as well as ethical failure. The one, perhaps, silver lining is that it has certainly energised politicians around the world, because it became headline news for weeks. Politicians took note and, in a lot of countries, that has led to legislation and regulations to try to prevent these kinds of things. As scientists, we're trying to do better going forward by being even more prescriptive, having more working groups and publishing even more massive documents. But unless everyone enforces these rules everywhere, there's nothing to stop someone going to a country where there is no legislation or regulation and doing these experiments.

**What do you enjoy doing outside of the lab and work?**

I have to confess, over the last few years, my hobby has been entrepreneurship. To be fair, it's entrepreneurship in the service of my research goal, so it's taken me outside of the lab in some ways, but it's still tied to the work that has been going on in my lab. It's been interesting just to learn about the process of starting companies and it has been fun engaging with investors, raising money and taking the company public. I view it as very orthogonal to my day job, so to me it feels like one big hobby that keeps me excited and engaged. I have other hobbies that I haven't had much time for over the last few years, but I'm doing what I love, and I'm learning so much, so what more can one ask for?
